# Regulation of the Phytoplankton Heme *b* Iron Pool During the North Atlantic Spring Bloom

**DOI:** 10.3389/fmicb.2019.01566

**Published:** 2019-07-11

**Authors:** Evangelia Louropoulou, Martha Gledhill, Thomas J. Browning, Dhwani K. Desai, Jan-Lukas Menzel Barraqueta, Manon Tonnard, Géraldine Sarthou, Hélène Planquette, Andrew R. Bowie, Ruth A. Schmitz, Julie LaRoche, Eric P. Achterberg

**Affiliations:** ^1^GEOMAR Helmholtz Centre for Ocean Research Kiel, Kiel, Germany; ^2^Institute for General Microbiology, Christian-Albrechts-Universität zu Kiel, Kiel, Germany; ^3^Department of Biology, Dalhousie University, Halifax, NS, Canada; ^4^Department of Earth Sciences, Stellenbosch University, Stellenbosch, South Africa; ^5^UMR 6539/LEMAR/IUEM, CNRS, UBO, IRD, Ifremer, Brest, France; ^6^Antarctic Climate and Ecosystems Cooperative Research Centre, Hobart, TAS, Australia; ^7^Institute for Marine and Antarctic Studies, University of Tasmania, Hobart, TAS, Australia

**Keywords:** heme *b*, North Atlantic, phytoplankton, diatoms, iron, limitation, GEOTRACES, GEOVIDE

## Abstract

Heme *b* is an iron-containing co-factor in hemoproteins. Heme *b* concentrations are low (<1 pmol L^-1^) in iron limited phytoplankton in cultures and in the field. Here, we determined heme *b* in marine particulate material (>0.7 μm) from the North Atlantic Ocean (GEOVIDE cruise – GEOTRACES section GA01), which spanned several biogeochemical regimes. We examined the relationship between heme *b* abundance and the microbial community composition, and its utility for mapping iron limited phytoplankton. Heme *b* concentrations ranged from 0.16 to 5.1 pmol L^-1^ (median = 2.0 pmol L^-1^, *n* = 62) in the surface mixed layer (SML) along the cruise track, driven mainly by variability in biomass. However, in the Irminger Basin, the lowest heme *b* levels (SML: median = 0.53 pmol L^-1^, *n* = 12) were observed, whilst the biomass was highest (particulate organic carbon, median = 14.2 μmol L^-1^, *n* = 25; chlorophyll *a*: median = 2.0 nmol L^-1^, *n* = 23) pointing to regulatory mechanisms of the heme *b* pool for growth conservation. Dissolved iron (DFe) was not depleted (SML: median = 0.38 nmol L^-1^, *n* = 11) in the Irminger Basin, but large diatoms (*Rhizosolenia* sp.) dominated. Hence, heme *b* depletion and regulation is likely to occur during bloom progression when phytoplankton class-dependent absolute iron requirements exceed the available ambient concentration of DFe. Furthermore, high heme *b* concentrations found in the Iceland Basin and Labrador Sea (median = 3.4 pmol L^-1^, *n* = 20), despite having similar DFe concentrations to the Irminger Basin, were attributed to an earlier growth phase of the extant phytoplankton populations. Thus, heme *b* provides a snapshot of the cellular activity *in situ* and could both be used as indicator of iron limitation and contribute to understanding phytoplankton adaptation mechanisms to changing iron supplies.

## Introduction

Iron (Fe) is an essential micro-nutrient for marine phytoplankton as it is associated with several key biochemical processes including photosynthesis, respiration, reduction of oxidized nitrogen species and di-nitrogen (N_2_) fixation (Geider and La Roche, 1994; Raven et al., 1999; Morel and Price, 2003). Iron uptake by phytoplankton occurs directly from seawater and the availability of Fe in the surface ocean is largely controlled by rates of supply from atmospheric deposition, continental margins, upwelling and deep mixing entrainment of sub-surface Fe pools, relative to losses via scavenging and biological uptake (Sunda and Huntsman, 1995; Geider, 1999; Tagliabue et al., 2017). Dissolved iron (DFe) concentrations in the open ocean surface waters can be very low (<0.1 nmol L^-1^) (Sunda and Huntsman, 1995; Boyd and Ellwood, 2010; Achterberg et al., 2018) and it has been well documented that low Fe availability can limit phytoplankton growth, N_2_ fixation rates, and influence community structure (e.g., Greene et al., 1992; Geider and La Roche, 1994; Kolber et al., 1994; Sunda and Huntsman, 1997; King and Barbeau, 2007; Shi et al., 2007).

In proteins, Fe is bound in Fe-sulfur clusters, Fe-oxygen-Fe clusters, or in Fe-porphyrin complexes also known as hemes (Hogle et al., 2014). Hemes function as prosthetic groups of the hemoproteins (Chapman et al., 1997) and are involved in electron transfers for example during photosynthesis, respiration, nitrate reduction as well as in control, storage and transport of oxygen (Hogle et al., 2014). Hemes are produced in a similar manner to chlorophyll via the tetrapyrrole synthesis pathway and several heme structures are present within an organism (Chapman et al., 1997). The cellular heme quota is tightly coordinated, since free hemes are toxic (Espinas et al., 2012). Furthermore heme can act as an Fe source for bacteria (Roe et al., 2013; Hogle et al., 2016).

Heme *b* (Fe protoporphyrin IX) is the most versatile heme in organisms (Espinas et al., 2012) and is a constituent of the *b* type cytochromes, catalases, peroxidases, cytochrome p450, globin and nitrate reductase (Mochizuki et al., 2010; Hogle et al., 2014). Culturing experiments involving eukaryotes and prokaryotes (*Dunaliella tertiolecta*, *Emiliania huxleyi*, *Thalassiosira oceanica*, *Synechococcus* sp.) have shown that heme *b* accounts for 18 ± 14% of total cellular Fe inventory (Honey et al., 2013).

The information regarding heme *b* abundance in the oceans is limited, with only a few studies that have reported heme *b* concentrations in surface waters of the Atlantic and Southern Oceans (Gledhill et al., 2013, 2015; Honey et al., 2013). Increased heme *b* concentrations were associated with high biomass in the North Atlantic and Southern Oceans (Gledhill et al., 2013). Recent work has also shown that the relative abundance of heme *b* and biomass stocks, as indicated by particulate organic carbon (POC) and chlorophyll *a* (chl *a*) concentrations, changes with bloom progression under Fe replete conditions (Bellworthy et al., 2017). Hence, heme *b* was observed to decrease rapidly post-bloom in a mesocosm experiment, although this effect might be dampened in open ocean environments by a lower Fe availability (Bellworthy et al., 2017). In Fe deplete regions, heme *b* concentrations decreased in marine particulate material (Honey et al., 2013) suggesting that heme *b* could potentially be used as a proxy for Fe limitation in field studies. Furthermore, fluctuations in heme *b* concentrations among different phytoplankton classes and species also imply that heme *b* abundance in the ocean could potentially vary according to the phytoplankton community composition (Gledhill et al., 2013, 2015; Honey et al., 2013).

Laboratory experiments performed on diatoms isolated from temperate and/or coastal regions (*Thalassiosira weissflogii*, *T. oceanica*, *Phaeodactylum tricornutum*, and *Chaetoceros calcitrans*) and cyanobacteria (*Synechococcus* sp.) showed that decreases in the heme *b* cellular contents were reflected in decreased biomass stocks under low DFe (≤0.5 nmol L^-1^) (Honey et al., 2013; Gledhill et al., 2015). In contrast, experiments on prymnesiophytes (*Emiliania huxleyi*, *Phaeocystis antarctica*) and diatoms (*Chaetoceros brevis*), all abundant in the open ocean, showed that these species were capable of maintaining their biomass stocks (e.g., growth rates, chl *a* and POC) despite reduced intracellular heme *b* concentrations at low DFe concentrations (≤0.5 nmol L^-1^) (Gledhill et al., 2015). These findings suggest that both diatoms and prymnesiophytes possess heme *b* regulation mechanisms which are expressed by a preferential allocation of Fe away from the hemoprotein pool in order to efficiently exploit the available Fe, reduce the overall Fe requirements and maintain growth (Gledhill et al., 2015). It is likely that such a regulation mechanism reflects an overall reduction in cellular Fe requirement until a subsistence Fe quota is obtained, below which growth can no longer occur.

In the current study we determined heme *b* in particulate material (>0.7 μm) sampled in the subtropical and subpolar North Atlantic Ocean during the GEOVIDE (GEOTRACES section G01) research expedition (Sarthou et al., 2018). The North Atlantic Ocean is an area of high phytoplankton productivity and enhanced CO_2_ sequestration rates (Henson et al., 2009). Several biogeochemical regimes exist in the North Atlantic Ocean; in the temperate subtropics, nitrate limits phytoplankton growth (Moore et al., 2008, 2013). At higher latitudes in the subpolar areas, during spring and summer, light is no longer a limiting factor for phytoplankton due to the lengthening of daylight period, while nitrate and phosphate are in excess (Henson et al., 2009; Harrison et al., 2013). However, DFe concentrations can become depleted (<0.1 nmol L^-1^) (Achterberg et al., 2018) and these regions can thus become Fe limited during late spring and summer months (Moore et al., 2008; Nielsdóttir et al., 2009). Specifically, Fe-limitation has been observed after the peak spring bloom in the Iceland Basin (Nielsdóttir et al., 2009; Ryan-Keogh et al., 2013; Macey et al., 2014), Irminger Basin (Sanders et al., 2005; Ryan-Keogh et al., 2013; Macey et al., 2014), and Labrador Sea (Harrison et al., 2013; Fragoso et al., 2017). Furthermore, shifts in phytoplankton assemblages in the high latitude North Atlantic during the spring-summer bloom are potentially induced by the individual requirements for Fe of each phytoplankton class (e.g., Boyd et al., 2012; Strzepek et al., 2012; Ryan-Keogh et al., 2013, 2017).

Our hypothesis was that the heme *b* abundance in the subtropical and subpolar North Atlantic Ocean would vary according to biomass, and that low heme *b* concentrations would be an indicator of low Fe regimes. A reduction in the ratios of heme *b* relative to biomass would imply optimization of the heme *b* – containing Fe pool by phytoplankton in order to maintain carbon fixation. Furthermore, we hypothesized that Fe concentrations at which optimization is observed would vary among phytoplankton groups and also depend on the stage of the bloom. Finally, this study also connects the heme *b* variability with data on phytoplankton community composition and thus contributes to our understanding of how available Fe is utilized by different phytoplankton groups.

## Materials and Methods

### Study Area and Field Sampling

**FIGURE 1:**
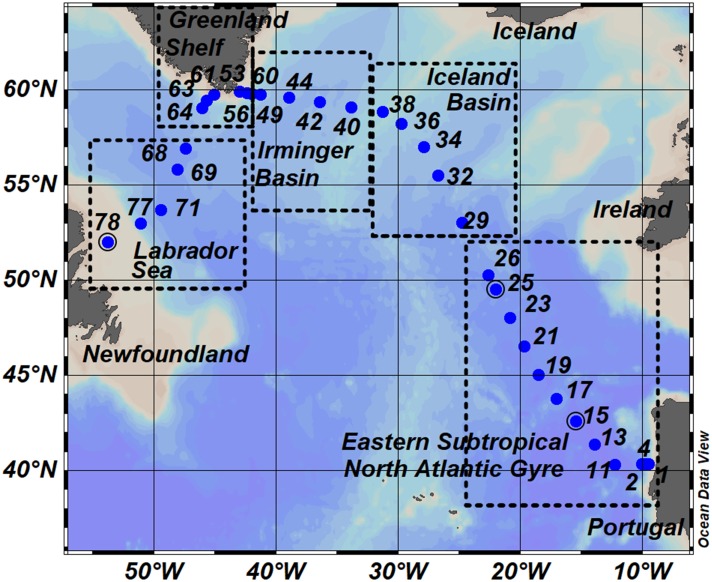
Map of sampling stations occupied during GEOVIDE Cruise (GEOTRACES – section GA01). Dots represent sampling stations and number annotations indicate the station identification numbers. The transect is divided into five sub-regions: the Eastern Subtropical North Atlantic Gyre (ESNAG), Iceland Basin (IceB), Irminger Basin (IrmB), the Greenland Shelf (GSH) and the Labrador Sea (LS).

We collected samples in the subpolar and subtropical North Atlantic Ocean during the GEOVIDE (GEOTRACES-GA01 section) research cruise which took place in May–June 2014. Discrete samples were collected from a total of 32 stations (Figure 1) located in the Eastern Subtropical North Atlantic Gyre (Stations 1–26), Iceland Basin (Stations 29–38), Irminger Basin (Stations 40–49 and 60), along the Greenland Shelf (Stations 53–56 and 61–64), and in the Labrador Sea (Stations 68–78).

### Temperature, Salinity and Mixed Layer Depth

Temperature and salinity data were obtained during the voyage using CTD sensors (SBE911 equipped with SBE-43) on a stainless steel hydrographic rosette frame. The surface mixed layer (SML) was calculated from the CTD data as described by Tonnard et al. (2018) applying the density criterion (Δσ_t_ = 0.125 kg m^-3^) with an increase in density relative to the surface density of less than Δσ_t_ for the whole mixed layer (Kara et al., 2000). We also calculated the SML using the temperature-based criterion (ΔT = 0.5°C) (Monterey and Levitus, 1997). Where a discrepancy was observed, we used the SML that most closely coincided with the nutricline and ferricline.

### Heme *b*

Seawater was sampled from up to 6 depths per station (<200 m). Particles were filtered onto glass fiber filters (nominal pore size 0.7 μm, Fisherbrand, MF300), and the filters were stored at -80°C prior to analysis.

For the determination of heme *b*, the method developed by Gledhill (2014) was followed with slight modifications. Briefly, 1 mL of the extraction solution consisting of 2.5% w:v Octyl β-D-Glucopyranoside-OGP (Sigma-Aldrich^®^, ≥98% GC Grade) in 0.02 mol L^-1^ NH_4_OH (HPLC Grade, Fisher) was added to each filter. Samples were vortexed for 10 s, ultrasonicated on ice for 45 s using an ultrasonic probe (Bandelin Sonopuls UW70, 20 kHz, 40% amplitude) and finally filtered using centrifuge tube filters (Corning^®^Costar^®^Spin-X^®^, cellulose acetate, 0.22 μm) for 10 min at 5°C at 6,800 ×*g*. In parallel, a set of standard solutions of Fe(III) protoporphyrin IX chloride [Fe(III)PTP] (Hemin, Frontier Scientific Discovery Chemicals) was prepared for calibrations. The concentration of the calibration standards ranged from 0 to 16.3 nmol L^-1^ Fe(III)PTP. Both the extraction solution and the calibration standards were made fresh daily due to the reduced chemical stability of OGP (Gledhill, 2007).

The quantification of heme *b* was performed using separation with High Performance Liquid Chromatography (PLRP-S column 2.1 × 100 mm, 300 Å, 3 μm, Agilent Technologies) and detection by Electrospray Ionization using a Q Exactive^TM^ Mass Spectrometer (Thermo Fisher Scientific). Conditions were similar to those previously described (Gledhill, 2014; Bellworthy et al., 2017) except for the following modifications. In this study, formic acid was used as a mobile phase modifier since our test runs indicated a more stable signal and reproducible results for consecutive days of analysis. The aqueous phase (Solvent A) consisted of isopropanol (Optima^®^LC/MS Grade, Fisher Chemical), acetonitrile (Optima^®^, LC/MS Grade, Fisher Chemical), Milli-Q and formic acid (Optima^®^, LC/MS Grade, Fisher Chemical) in a ratio 0.5:0.5:90:0.1% (v:v:v:v) whereas the organic phase (Solvent B) consisted of a mixture of isopropanol (Optima^®^LC/MS Grade, Fisher Chemical), acetonitrile (Optima^®^LC/MS Grade, Fisher Chemical), and formic acid (Optima^®^, LC/MS Grade, Fisher Chemical) in a ratio 50:50:0.1% v:v:v. The flow rate of the HPLC was 400 μL min^-1^ and the starting conditions of the HPLC were 60% solvent A: 40% solvent B with a linear gradient increase of Solvent B to 100% over 10 min. The column temperature was 21°C.

In this study the high resolution (70,000 at m/z = 200) ESI-MS Orbitrap detector allowed for quantification of heme *b* using the exact mass of the M^+^ ion (m/z = 616.177). The injection volume was 25 μL and the heme *b* peak eluted between 2.0 and 3.0 min. The higher resolution (5 ppm) of the mass spectrometer eliminated isobaric interferences encountered with previous MS analysis (Gledhill, 2014). The analytical detection limit for 25 μL injection volume was calculated to be 32 pmol heme *b* L^-1^ determined as three times the standard deviation of the lowest standard (4.1 nmol heme *b* L^-1^). This detection limit does not account for the pre-concentration factor resulting from filtration and extraction. Taking into account the pre-concentration factor, results in detection limits ranging from 0.01 to 0.02 pmol L^-1^ of heme *b* for field samples. Finally, heme *b* could not be detected in the blank extraction solution.

### Microbial Community Composition

DNA samples were collected by vacuum (20 to 30 kPa) filtering 2 L of seawater onto a polycarbonate filter (Millipore Isopore 0.2 μm 47 mm – GTTP04700) and stored in a cryovial at -80°C until analysis in the laboratory.

DNA was extracted using the QIAGEN DNeasy Plant Mini Kit as directed by the manufacturer, with the following modification to improve cell lysis at the initial step of the procedure. Briefly, 50 μL of lysozyme solution (5 mg mL^-1^ in TE buffer), 45 μL of Proteinase K solution [20 mg mL^-1^ in deionized (Milli-Q) PCR grade water] and 400 μL of AP1 lysis buffer from the QIAGEN DNeasy Plant Mini Kit were added to the filter in the cryovial and incubated at 52°C on an orbital shaker (300 rpm) for 1 h before continuing as directed by the manufacturer. DNA concentration and purity were assessed with NanoDrop 2000 (Thermo Fisher Scientific, United States) and then stored at -80°C.

The microbial community was characterized by next generation sequencing (NGS) of the 16S rRNA gene V6–V8 variable region on an Illumina MiSeq instrument, following the Microbiome Amplicon Sequencing Workflow (Comeau et al., 2017). Samples were amplified using dual-indexing Illumina fusion primers that targeted the 438 bp V6–V8 region with the forward and reverse primers B969F 5′-ACGCGHNRAACCTTACC and BA1406R 5′-ACGGGCRGTGWGTRCAA (Comeau et al., 2012).

The DNA reads from each sample were processed using the QIIME pipeline version 1.8.0 (Caporaso et al., 2010b) following an established workflow (Comeau et al., 2017). Paired-end sequences were demultiplexed and merged by PEAR version 0.9.6 (Zhang et al., 2014). Sequences less than 400 bp in length or with a quality less than 30 over 90% of bases were discarded and VSEARCH was used to remove chimeric sequences (Rognes et al., 2016). The quality-controlled paired reads were assigned to operational taxonomic units (OTUs) by clustering at the 97% similarity using *sortmerna* (Kopylova et al., 2012) for reference picking using the Greengenes version 13.8 database (McDonald et al., 2012). *De novo* picking of reads not assigned with Greengenes was carried out with *sumaclust* (Mercier et al., 2013). Singletons and low-confidence OTUs were removed using PyNAST (Caporaso et al., 2010a). Chloroplast (cp) 16S rRNA gene sequences were further classified using the PhytoRef database (Decelle et al., 2015).

The relative abundance of taxa was based on an OTU matrix rarified at 1290 reads for bacterial 16S rRNA and 100 reads cp 16S rRNA sequences, in order to allow meaningful comparison of the community structure that included all of the relevant samples. All 16S rRNA gene sequences are available on the Supplementary Data Sheet 1. The accession numbers of the cp 16S rRNA sequences are presented on Supplementary Table 2.

### Supporting Variables

We collected discrete samples of seawater from the CTD rosette or trace metal rosette (TMR) from different depths for the determination of nitrate, particulate organic carbon (POC), chlorophyll *a* and dissolved Fe (DFe).

Nitrate concentrations were measured using standard colorimetric techniques with an auto-analyzer as described by Aminot and Kerouel (2007) and are published in Sarthou et al. (2018). Phytoplankton chlorophyll *a* was determined after extraction with 100% methanol by HPLC analysis following the protocol of Ras et al. (2008) and details are published in Tonnard et al. (2018).

Seawater was filtered for the determination of POC on pre-combusted glass fiber filters (MF300, Fisherbrand) which were rinsed with deionized water to remove salts and then dried at 60°C. Data are published in Sarthou et al. (2018). Prior to analysis, dried filters were fumed with hydrochloric acid to remove inorganic carbon. The POC was determined using an elemental analyzer (ThermoQuest, Flash 1112 series) as described in Lorrain et al. (2003).

The method used for analysis of DFe is described in Tonnard et al. (2018). Briefly, seawater was sampled using a trace metal clean polyurethane powder-coated aluminum frame rosette (TMR) equipped with 12 L, Teflon lined GO-FLO bottles. Seawater samples were analyzed for DFe using a SeaFAST-pico^TM^ coupled to an Element XR HR-ICP-MS (Tonnard et al., 2018).

### Data Handling

The Mass Spectrometry data were processed using the Thermo Xcalibur^TM^ Software v.3.0.33. Section and contour plots were produced using the Ocean Data view v.4.7.9. software (Schlitzer, 2018). Further data processing and visualization were performed by R Statistical Software (R Core Team, 2016). The Shapiro-Wilk test was performed to check the normality of the distributions and indicated non-parametric distribution for the whole dataset (*p* < 0.05). The association between parameters was determined using the Spearman’s rank correlation coefficient. Finally, the non-parametric ANOVA Kruskal-Wallis and the *post hoc* Dunn’s test were used to identify significant differences among the sub-regions of the transect. Due to the non-normal distribution of the dataset, the ranges and the median values of each parameter are reported.

## Results and Discussion

### Sampling Region and Hydrography

**FIGURE 2:**
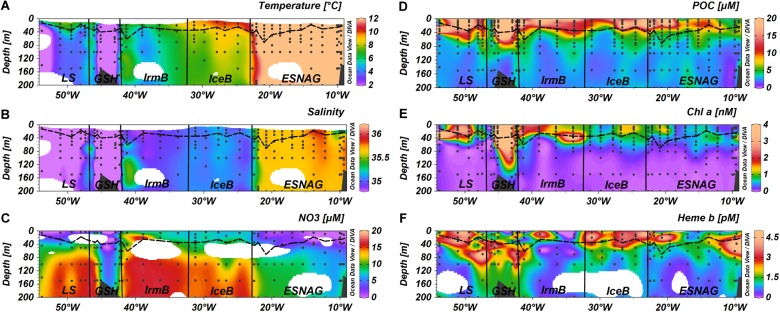
Section plots of **(A)** Temperature, **(B)** Salinity, **(C)** Nitrate, **(D)** Particulate Organic Carbon (POC), **(E)** chlorophyll *a* (Chl *a*), and **(F)** heme *b* across the GEOVIDE transect. Dashed lines indicate the Mixed Layer Depth and dots indicate the stations and depths where samples were taken. Letter annotations indicate the five oceanographic regions; Eastern Subtropical North Atlantic Ocean (ESNAG), Iceland Basin (IceB), Irminger Basin (IrmB), Greenland Shelf (GSH), and Labrador Sea (LS).

Temperature and salinity profiles of the section for the top 200 m of the water column are presented in Figure 2. Regarding the surface waters, the North Atlantic Current (NAC) carries warm and saline water in the Eastern North Atlantic. The NAC causes the formation of the Subarctic Front between stations 26 and 29 which separates the warm waters of the subtropical gyre with colder waters in the subpolar gyre. Following a cyclonic circulation, the NAC feeds the Irminger current (IC) that flows west of the Reykjanes Ridge into the Irminger Basin. The East Greenland current (EGC) carries cold and fresh Arctic water southwards around the Greenland Shelf into the Labrador Sea and finally merges with the Labrador current (LC) flowing west in the subpolar gyre.

The depths of the surface mixed layer (SML) were chosen according to a density criterion, however, for station 63 on the Greenland Shelf we selected the temperature criterion since the depth coincided better with the nutricline and the ferricline. Overall, the SML depth ranged between 13.4 and 69.5 m with a median value of 33.5 m across the section. There were no significant differences for the SML depth among the sub-regions of the section (Kruskal-Wallis-Test, *p* > 0.05).

We divided our dataset between samples located within the SML and those below it. Furthermore, we also defined five oceanographic regions (Figure 1) according to the geographical position of the stations, temperature, salinity and nitrate distributions (Figure 2). The five oceanographic regions consist of the Eastern Subtropical North Atlantic Gyre (ESNAG), the Iceland Basin (IceB), the Irminger Basin (IrmB), the Greenland Shelf (GSH), and the Labrador Sea (LS).

### Nitrate and Dissolved Iron Distributions

**FIGURE 3:**
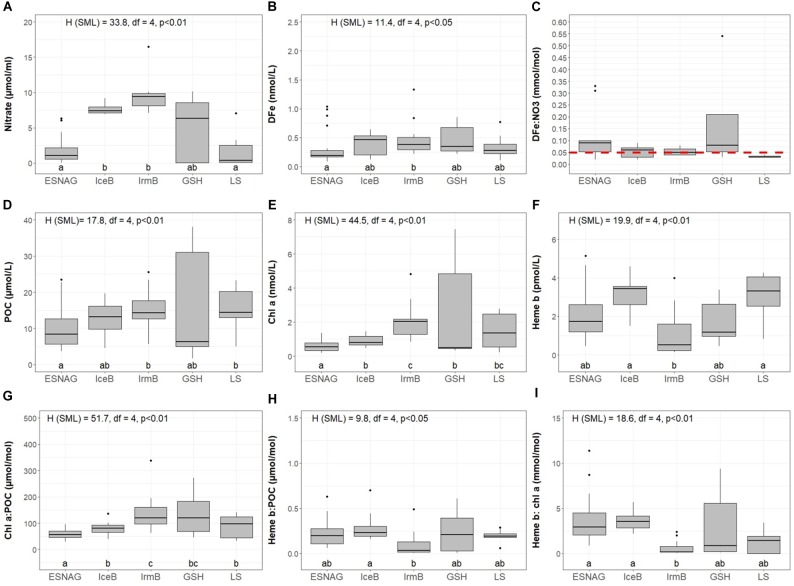
Boxplots of distributions in the surface mixed layer (SML) of **(A)** Nitrate, **(B)** Dissolved iron (DFe), **(C)** DFe:nitrate, **(D)** Particulate Organic Carbon (POC), **(E)** Chlorophyll *a* (Chl *a*), **(F)** Heme *b*, **(G)** Chl *a*:POC, **(H)** Heme *b*:POC, and **(I)** Chl *a*:heme *b* for the five oceanographic regions; Eastern Subtropical North Atlantic Ocean (ESNAG), Iceland Basin (IceB), Irminger Basin (IrmB), Greenland Shelf (GSH), and Labrador Sea (LS). On top of sub-plots **A, B, D, E, F, G, H**, and **I** the result of the Kruskal-Wallis test is annotated. The dots above the boxes indicate the outliers. Letters (letters a to c) below the boxes indicate the Compact Letter Display (cld) of the statistically significant different groups in the SML after a *Post hoc* test for multiple comparisons of groups. The red dashed line in plot C marks the threshold value 0.01 mmol mol^-1^ for the assessment of Fe-stressed phytoplankton (Sunda and Huntsman, 1995; Ho et al., 2003; Nielsdóttir et al., 2012).

Nitrate concentrations were low in the SML of the ESNAG (median = 1.6 μmol L^-1^, *n* = 19) and the LS (median = 0.23 μmol L^-1^, *n* = 6) and intermediate on the GSH (median = 5.06 μmol L^-1^, *n* = 7) (Figure 3A). Nitrate was elevated in IceB and IrmB exhibiting median concentrations of 7.39 μmol L^-1^ (*n* = 10) and 9.40 μmol L^-1^ (*n* = 9), respectively. The depth distribution of nitrate (Figure 2C) indicated a clear nutricline at approximately 50 m depth for the subtropical areas and 40 m for subpolar areas.

Dissolved Fe (DFe) ranged from 0.09 to 2.14 nmol L^-1^ (median = 0.41 nmol L^-1^, *n* = 59) (Figure 3B) in the SML across the transect (Tonnard et al., 2018). The spatial distribution of DFe indicated statistically significant lower concentrations (<0.5 nmol L^-1^) within the ESNAG compared to the subpolar areas (IceB, IrmB, GSH, and LS). Higher DFe concentrations (>0.75 nmol L^-1^) were also determined close to the continental margins (Iberian and GSH). Furthermore, the concentrations of DFe during the time of our study in the IrmB (SML: range 0.22 to 1.33 nmol L^-1^, median = 0.38 nmol L^-1^, *n* = 11) were elevated compared to observations obtained in the spring (April-May, mean = 0.124 ± 0.06 nmol L^-1^, *n* = 21) and summer (mean = 0.087 ± 0.234 nmol L^-1^, *n* = 48) in 2010 (Achterberg et al., 2018).

The ratio of DFe:nitrate ranged from 0.02 to 38.6 mmol mol^-1^ (Tonnard et al., 2018) overall along the transect, with the lowest values in the IceB and IrmB (mean = 0.05 mmol mol^-1^) (Figure 3C).

### Particulate Organic Carbon and Chlorophyll *a* as Biomass-Indicative Parameters

The section of POC showed enhanced concentrations in the SML for most regions (Figure 2D, 3D). Overall, POC ranged from 1.72 to 40.0 μmol L^-1^ in the SML (median = 11.6 μmol L^-1^, *n* = 140). The lowest concentrations of POC were observed in the ESNAG, where the median value was 8.45 μmol L^-1^ in the SML (*n* = 52) and 3.09 μmol L^-1^ below the SML (*n* = 80). Statistically significant differences were observed for POC among the five oceanographic regions according to the Kruskal-Wallis-Test (Figure 3D). A pairwise comparison showed that they were attributed mainly to differences between the low biomass ESNAG and the remaining regions (Figure 3D).

Figure 2E illustrates the section for chl *a*, with concentrations ranging from 0.14 to 7.44 nmol L^-1^ within the SML (median = 0.71 nmol L^-1^, *n* = 134). The highest concentrations in the SML were observed in the high latitude regions of IrmB, GSH and LS (Median = 1.66 nmol L^-1^, *n* = 61), whereas lower chl *a* was measured in the ESNAG and the IceB (median = 0.60 nmol L^-1^, *n* = 70). Statistically significant differences were observed for chl *a* between the oceanographic regions (Figure 3E, Kruskal-Wallis, *p* < 0.01). Chlorophyll *a* correlated well with POC (Spearman’s rho, *r* = 0.88, *n* = 296, *p* < 0.01) (Supplementary Table 1).

Stratification of the water column can lead to the formation of a Deep Chlorophyll Maximum (DCM) in oligotrophic waters and post-bloom during the summer in temperate (Cullen, 1982; Hickman et al., 2012; Mignot et al., 2014 etc.) and Polar regions (Holm-Hansen and Hewes, 2004). A DCM was observed in the oligotrophic ESNAG and in the GSH at approximately 60 m depth, in IrmB (station 40) at 40 m depth and in the Newfoundland Shelf (station 78) of the LS (Figure 2E).

### Heme *b* Concentrations and Distribution

The vertical distribution of heme *b* (Figure 2F) indicated enhanced concentrations in the SML, which decreased in general with depth, with the exception of stations where a DCM was present. Overall heme *b* concentrations ranged from 0.16 to 5.13 pmol L^-1^ (median = 2.17 pmol L^-1^, *N* = 65) within the SML. Below the SML (down to 200 m depth), the median concentration of heme *b* was 1.14 pmol L^-1^ (*N* = 107). In the DCM of the ESNAG and GSH, heme *b* reached concentrations up to 5.67 pmol L^-1^, whereas no increase was observed in the DCMs of the IrmB and LS. Across the section, the lowest concentrations of heme *b* were detected in the IrmB (SML: median = 0.53 pmol L^-1^, *n* = 12), and the Kruskal-Wallis-Test confirmed a statistically significant difference in the distribution of heme *b* among the sub-regions and particularly between the IrmB and IceB (Figure 3F).

Our results were generally consistent with previously published data (Table 1). In 2010, heme *b* averaged 1.3 ± 0.60 pmol L^-1^ in the subtropical North Atlantic (Honey et al., 2013), which is comparable to the average of 1.5 ± 1.2 pmol L^-1^ that we determined in our study for the same region. However, heme *b* concentrations in the IceB were slightly higher during the GEOVIDE cruise (mean = 2.1 ± 1.1 pmol L^-1^) compared to data from July–August 2007 (mean = 1.1 ± 0.7 pmol L^-1^) (Gledhill et al., 2013) (Table 1), which is probably due to the sampling in different seasons (spring vs. late summer) and to the different phytoplankton bloom stages.

**Table 1:** Comparison table of heme *b*, chlorophyll *a* and dissolved iron concentrations observed in the North Atlantic Ocean (May–June 2014) to date.

Study area	Month/Year	Heme *b* (pmol L^-1^)	Chlorophyll *a* (nmol L^-1^)	Dissolved iron (nmol L^-1^)	References
Celtic Sea	July–August 2005	3.8 ± 1.7	0.5 ± 0.3	0.8–2.1	Ussher et al., 2007; Honey et al., 2013
Iceland Basin	July–August 2007	1.1 ± 0.7	1.2 ± 0.8	<0.03–0.22	Nielsdóttir et al., 2009; Gledhill et al., 2013
Scotia Sea	January–February 2008	5.1 ± 4.8	1.9 ± 1.7	<0.03–0.6	Nielsdóttir et al., 2012; Gledhill et al., 2013
Tropical North Atlantic	January–February 2008	2.3 ± 1.7	0.25 ± 0.13	<0.1–0.37	Rijkenberg et al., 2012; Gledhill et al., 2013
Subtropical North Atlantic	January–February 2010	1.3 ± 0.60	0.17 ± 0.11	NA	Honey et al., 2013
Gullmar Fjord	March–June 2013	66 ± 45	1.2 ± 0.6	4–40	Stolpe and Hassellöv, 2009; Bellworthy et al., 2017
Eastern Subtropical North Atlantic Gyre	May–June 2014	1.44 ± 1.12	0.30 ± 0.27	0.12-1.04	Current study
Eastern North Atlantic	May–June 2014	1.62 ± 1.27	0.49 ± 0.41	0.09-0.75	Current study
Iceland Basin	May–June 2014	2.09 ± 1.14	0.50 ± 0.37	0.12-2.23	Current study
Irminger Basin	May–June 2014	1.07 ± 1.00	1.10 ± 1.07	0.22-1.33	Current study
Greenland Shelf	May–June 2014	2.04 ± 1.15	1.66 ± 2.21	0.22-1.39	Current study
Labrador Sea	May–June 2014	2.16 ± 1.50	1.08 ± 1.79	0.11-0.77	Current study

*The data here are reported chronologically based on the sampling year. Adapted from Bellworthy et al. (2017).*

Heme *b* correlated weakly with both POC (Spearman’s rho, *r* = 0.41, *n* = 165, *p* < 0.01) and chl *a* (Spearman’s rho, *r* = 0.41, *n* = 153, *p* < 0.01) (Supplementary Table 1), in agreement with previous observations (Gledhill, 2007; Gledhill et al., 2013, 2015; Honey et al., 2013). Heme *b* did not exhibit any statistically significant relationship with nitrate or DFe (Supplementary Table 1).

### Ratios of Heme *b*, Chlorophyll *a* and Particulate Organic Carbon

We calculated the ratios of heme *b*:POC, chl *a*:POC and heme *b*: chl *a* in order to examine how heme *b* and chl *a* abundances changed relative to biomass. Heme *b*:POC and chl *a*:POC ratios represent, respectively, the heme *b* and chl *a* per unit carbon derived from the total organic matter (i.e., phytoplankton, zooplankton, bacteria, detritus). In addition, we used the ratio heme *b*: chl *a* to avoid conflation by zooplankton and dead matter since it is a proxy for the biomass of phytoplankton only.

The chl *a*:POC ratio (Figure 3G) was comparable in the ESNAG and IceB where it ranged from 29.0 to 137 μmol mol^-1^ in the SML (median = 61.4 μmol mol^-1^, *n* = 65). Prominent variations in the ratio were observed for the IrmB, GSH and LS where the chl *a*: POC ranged from 32.1 to 339 μmol mol^-1^ (median = 115 μmol mol^-1^, *n* = 61). Enhanced chl *a*: POC ratios were also observed in the DCMs (depth down to 80 m; range: 6.8 to 412 μmol mol^-1^, median = 92.0 μmol mol^-1^) (Supplementary Figure 1A). Increases in the chl *a*: POC ratios in the SML of the IrmB, GSH and LS and in the DCMs pointed to low-light acclimated phytoplankton communities in the subpolar regions (Henson et al., 2009; Harrison et al., 2013; Fragoso et al., 2016, 2017) and below the surface waters, respectively.

The heme *b*:POC ratio in the SML (Figure 3H) was significantly (Kruskal-Wallis test, *H* = 9.8, *p* < 0.05) lower in IrmB (median = 0.04 μmol mol^-1^, *n* = 12) and intermediate in ESNAG, GSH and LS (median = 0.19 μmol mol^-1^, *n* = 40) when compared to IceB (median = 0.28 μmol mol^-1^, *n* = 7). Furthermore, the sections of heme *b*:POC showed an increase in the ratio in the DCMs (median = 0.30 μmol mol^-1^, *n* = 50) (Supplementary Figure 1B). The heme *b:* chl *a* ratio followed a similar trend as the heme *b*:POC ratio (Figure 3I) and was lowest in IrmB (median = 0.19 mmol mol^-1^, *n* = 11), intermediate in GSH and LS (median = 1.45 mmol mol^-1^, *n* = 17) and significantly higher in ESNAG and IceB (median = 3.10 mmol mol^-1^, *n* = 30).

In general, the correlation of heme *b* with POC suggested that changes in both vertical and spatial distributions of heme *b* were driven primarily by changes in biomass, as reported previously (Gledhill et al., 2013; Honey et al., 2013; Bellworthy et al., 2017). Hence, higher biomass resulted in higher heme *b* concentrations. The high variability in POC, chl *a* and heme *b* and their ratios on the GSH suggested fluctuating biomass linked to submesoscale processes in these coastal waters (Thomas et al., 2013; Swart et al., 2015; Hopwood et al., 2018). Heme *b* deviated from the biomass-driven distribution in two cases; in the DCMs and the IrmB (Figure 2).

Regarding the DCMs, heme *b*: POC ratios increased and followed a similar trend as chl *a*:POC thus indicative of low-light acclimated cells (Moore et al., 2006b) (Supplementary Figure 1B). Heme *b* is contained in the *b*-type hemoproteins present in the photosystems (i.e., cytochromes *b*_559_ and *b*_6_) (Hogle et al., 2014). Hence, the changes in the photosynthetic apparatus due to light availability are also reflected by changes in the vertical distribution of heme *b* (Honey et al., 2013) independent of biomass. However, the light-driven adaptation of heme *b*: POC was not observed in IrmB; heme *b*:POC exhibited its lowest values while chl *a*: POC increased. The increasing low-light acclimation and chl *a* concentrations in the high latitude IrmB were not accompanied by general increases in the proteins of the photosynthetic apparatus (e.g., heme *b*).

The IceB and IrmB showed statistically significant contrasts in heme *b*: POC and heme *b*:chl *a* ratios. In IceB, heme *b*:POC exhibited a median ratio of 0.28 μmol mol^-1^ (*n* = 7) and heme *b*:chl *a* median ratio of 3.6 mmol mol^-1^ (*n* = 11) in the SML. In contrast, low heme *b*: POC (<0.1 μmol mol^-1^) and heme *b*:chl *a* (<1 mmol mol^-1^) ratios characterized the IrmB, with previous studies reporting values in the same range for Fe-limited field and cultured phytoplankton (Gledhill et al., 2013, 2015; Honey et al., 2013). This contrasting behavior between the IrmB and the IceB is particularly noteworthy since the DFe (IceB; median = 0.52 nmol L^-1^, IrmB; median = 0.38 nmol L^-1^) and nitrate concentrations (IceB; median = 7.4 μmol L^-1^, IrmB; median = 9.4 μmol L^-1^) (Figure 3B) were similar in the two regions. Furthermore, both regions had DFe:nitrate ratios of <0.05 mmol mol^-1^ indicative of Fe limitation, since values between 0.05 and 0.9 mmol mol^-1^ are considered to be optimal for nutrient replete growth (Sunda and Huntsman, 1995; Ho et al., 2003; Twining et al., 2004). In the past, both the IceB and IrmB have been shown to be seasonally Fe limited following the spring bloom (Nielsdóttir et al., 2009; Ryan-Keogh et al., 2013; Macey et al., 2014), exhibiting similarly low DFe:nitrate with residual nitrate concentrations. Although these studies were undertaken during the late summer after the bloom peak (Nielsdóttir et al., 2009; Poulton et al., 2010; Mohamed et al., 2011; Moore et al., 2013; Ryan-Keogh et al., 2013; Macey et al., 2014; Achterberg et al., 2018), suboptimal supply of Fe relative to nitrate during winter overturn has been hypothesized to drive the onset of seasonal Fe limitation in IceB and IrmB.

In our study, the absolute concentrations of DFe and nitrate in IceB and IrmB suggested that both systems were DFe and nitrate replete. Therefore, we hypothesized that the contrast in heme *b* and its ratios arose because of different dominant phytoplankton groups. Furthermore, given that the bloom in the subpolar North Atlantic progresses from east to west (Olsen et al., 2008), such differences in heme *b* may also be attributed to different phases of the spring bloom between the two regions.

### Linking Heme *b* Concentrations With Microbial Community Composition

**FIGURE 4:**
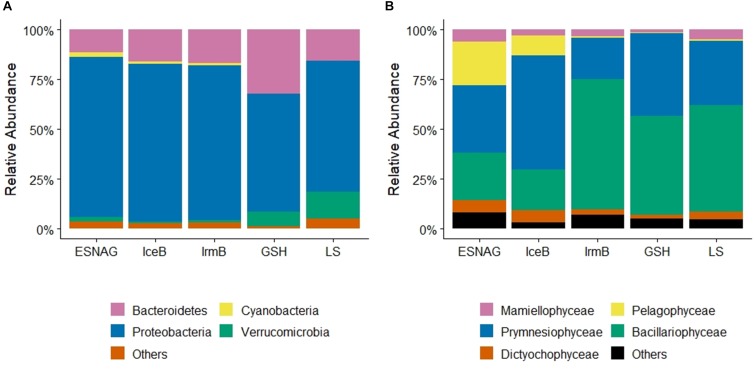
Relative abundance of **(A)** Prokaryotes, and **(B)** Eukaryotes across the GEOVIDE transect in the five oceanographic regions; Eastern Subropical North Atlantic Ocean (ESNAG), Iceland Basin (IceB), Irminger Basin (IrmB), Greenland Shelf (GSH), and Labrador Sea (LS).

Sequencing and analysis of the 16S rRNA and cp16S rRNA showed the relative abundance of prokaryotes (Figure 4A) and photosynthetic eukaryotes (Figure 4B) along the GEOVIDE section. Proteobacteria made up most of the prokaryotic pool (35–93%, median = 74%, *n* = 283) across the five oceanographic regions in the upper 100 m. Among the proteobacteria, alphaproteobacteria (mean = 84%, *n* = 283) were mostly abundant in all regions with representative species the *Pelagibacter* sp. Gammaproteobacteria accounted for 15% (mean, *n* = 283) of the proteobacteria pool. Cyanobacteria ranged from 0 to 11% (mean = 1.1%, *n* = 283) overall, but were mostly present in the ESNAG (Mean = 2.3%, *n* = 85). Since prokaryotes have only a small contribution to the total heme *b* pool (Gledhill, 2007), we proceeded with further data processing using the eukaryotes’ composition.

Mixed phytoplankton communities were present in all five oceanographic regions (Figure 4B). Overall, prymnesiophyceae (Mean = 36%, *n* = 245), bacillariophyceae (diatoms) (Mean = 40%, *n* = 245) and pelagophyceae (Mean = 8%, *n* = 245) were most abundant in the upper 100 m during the GEOVIDE expedition (May–June 2014). However, the percentage of each class varied among the regions (Figure 4B). In the ESNAG, pelagophyceae and prymnesiophyceae were the most abundant classes. In IceB, prymnesiophyceae dominated the eukaryotic phytoplankton community whereas in IrmB, GSH and LS bacillariophyceae were most abundant.

**FIGURE 5:**
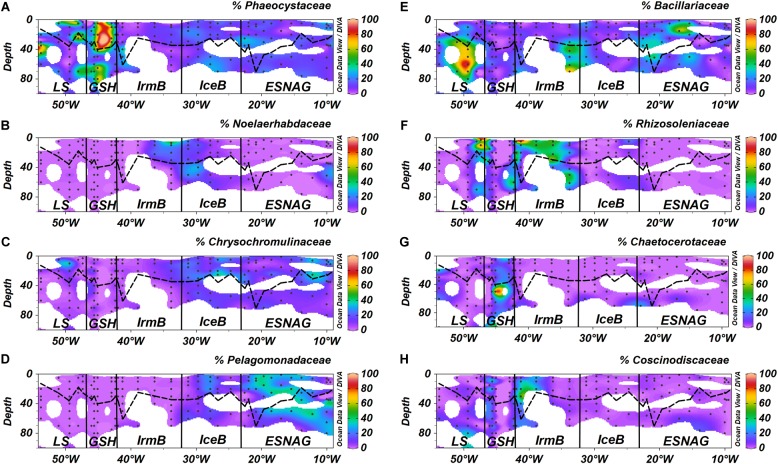
Section plots of relative abundance of major phytoplankton eukaryotic families **(A)** Phaeocystaceae, **(B)** Noelaerhabdaceae, **(C)** Chrysochromulinaceae, **(D)** Pelagomonadaceae, **(E)** Bacillariaceae, **(F)** Rhizosoleniaceae, **(G)** Chaetocerotaceae, and **(H)** Coscinodiscaceae across the GEOVIDE transect. Dashed lines indicate the Mixed Layer Depth and dots indicate the stations and depths where samples were taken. Letter annotations indicate the five oceanographic regions; Eastern Subropical North Atlantic Ocean (ESNAG), Iceland Basin (IceB), Irminger Basin (IrmB), Greenland Shelf (GSH), and Labrador Sea (LS).

More detailed analysis of the sequences allowed for a more precise taxonomic identification of the dominant phytoplankton families and species in each oceanographic region. Concerning the prymnesiophyceae, the most abundant families were Phaeocystaceae (Figure 5A), Noelaerhabdaceae (Figure 5B) and Chrysochromulinaceae (Figure 5C). The *Phaeocystis sp.* dominated the LS and the GSH while the *Emiliania* sp. (Noelaerhabdaceae) was mostly abundant in the eastern part of IrmB and in IceB. *Chrysochromulina* sp. were detected in ESNAG, IceB and LS. We also identified several families and species of bacillariophyceae (diatoms), including the Bacillariaceae (Figure 5E), the Rhizosoleniaceae (Figure 5F), the Chaetocerotaceae (Figure 5G), and the Coscinodiscaceae (Figure 5H) families. The large diatoms *Rhizosolenia* sp. (e.g., diameter = 4–20 μm, Length = 100–500 μm) and *Coscinodiscus* sp. (e.g., diameter 30–500 μm) were dominant in the IrmB while the smaller Bacillariaceae and Chaetocerotaceae were present in the other four oceanographic regions (ESNAG, IceB, GSH and LS). Finally, the Pelagomonadaceae (Figure 5D) family was present mostly in the ESNAG (*Pelagomonas* sp.).

Heme *b* depletion relative to biomass in IrmB thus points to an Fe-limited diatom-dominated phytoplankton community. In culture, both diatoms and prymnesiophytes can respond to Fe limitation by reducing heme *b* quotas (Honey et al., 2013; Gledhill et al., 2015). Despite the low heme *b* in IrmB, the biomass stocks (as indicated by POC and chl *a*) remained elevated pointing to regulation mechanisms of the hemoprotein pool which were likely employed by phytoplankton in order to efficiently reallocate the available Fe and conserve growth under the suboptimal DFe:nitrate conditions.

### Heme *b* Depletion Due to Phytoplankton Fe – Requirements

Diatoms are known for a high Fe demand (Sunda and Huntsman, 1995; Sarthou et al., 2005; Boyd et al., 2012) because of their relatively low surface area to volume ratio (Boyd et al., 2000; Timmermans et al., 2001b), while smaller-sized phytoplankton like prymnesiophytes have lower Fe requirements (Sunda et al., 1991; Sunda and Huntsman, 1995; Sunda and Hardison, 2010). Indeed half-saturation constants for Fe uptake (K_m_), indicating the external concentration of Fe required to support growth, range between 0.00059 to 1.2 nmol L^-1^ (Mean = 0.35 ± 0.44 nmol L^-1^) with larger diatom species exhibiting higher *K*_m_-values (Timmermans et al., 2001a, 2004; Sarthou et al., 2005). In contrast, the *K*_m_-value for the prymnesiophytes *Phaeocystis* sp. range from 5.0 to 258 pmol L^-1^ DFe (Coale et al., 2003). Furthermore, the degree of Fe stress varies among phytoplankton classes, with smaller sized phytoplankton consistently found to experience less severe Fe stress (Cullen, 1991; Price et al., 1994; Ryan-Keogh et al., 2013; Macey et al., 2014).

Iron and nitrate availabilities are higher at the beginning of the growing season (March–June) in the subpolar North Atlantic following Fe supply through deep convective mixing in winter, and the faster growing diatoms are first to bloom (Lochte et al., 1993). Later in the season when Fe concentrations decline, the bloom shifts to prymnesiophytes due to their lower Fe-demand (Lochte et al., 1993; Strzepek et al., 2011, 2012, Ryan-Keogh et al., 2013, 2017). Further subsequent decline of the available Fe during the bloom progression could then lead to Fe limiting conditions for the prymnesiophytes.

The contrast in heme *b* between the IrmB and the IceB in our study likely arises because of the individual Fe requirements and the growth phase of the extant phytoplankton groups. Therefore, the large diatom dominated IrmB (e.g., *Rhizosolenia* sp. size typically 100 – 500 μm length and 4 – 20 μm width) showed signs of Fe limitation despite relatively elevated Fe concentrations (SML: median = 0.38 nmol L^-1^, *n* = 11) because of their higher Fe-requirements compared to the prymnesiophytes (*Phaeocystis*, *Emiliania*, and *Chyrsochromulina* spp.) in the IceB (Sunda and Huntsman, 1995). In contrast, the haptophytes of IceB had not yet reached Fe -limited conditions at the time of the sampling (May–June 2014), probably because they were at an earlier growth stage compared to the diatoms in the IrmB (Henson et al., 2006).

We suggest that a reduction in heme *b* quota represents a general response of phytoplankton to the declining Fe concentrations over the course of the high latitude North Atlantic growth season, since it has now been observed in the field for both diatom (current study) and prymnesiophyte (Poulton et al., 2010; Gledhill et al., 2013) (Table 1). The reduction in heme *b* quota could indicate heme *b* regulation, driven by a suboptimal Fe supply and characterized by depletion relative to biomass. Regulation potentially occurs in both the declining diatom and prymnesiophyte populations but at a different time point with respect to ambient Fe concentrations as a result of differences in the absolute Fe requirements of each phytoplankton class. We suggest that once the subsistence heme *b* quotas (and related Fe quota) can no longer be supported, the extant population becomes Fe limited. The whole community will become progressively limited only when the species with the lowest Fe requirement can no longer satisfy its subsistence Fe quota. However, to fully establish the patterns of heme *b* abundance and their relationship to community composition and Fe availability relative to other nutrients during the bloom progression determination of heme *b* in concert with community composition over the course of an open ocean phytoplankton bloom is required.

Heme *b* regulation mechanisms likely include reduction in the abundance of the heme *b*-containing (Hogle et al., 2014) cytochromes *b_6_f* and *b_559_* of the PSII apparatus (Greene et al., 1992; Strzepek and Harrison, 2004) which in turn result in increases in chl *a*:PSII ratios as observed later in the growth season in the IrmB (Macey et al., 2014). Indeed, transcriptomic and proteomic analysis in other diatom species (*Thalassiosira pseudonada*, *T. oceanica*, and *Pseudo-nitzschia granii*) showed downregulation of the heme *b*-containing cytochrome *b_6_f* and conservation of the PSII proteins expression (Lommer et al., 2012; Nunn et al., 2013; Cohen et al., 2018). Another regulatory mechanism of heme *b* could be linked to the switch from nitrate to ammonium utilization (Price et al., 1991; Timmermans et al., 1994) which would in turn lead to decreases in the heme *b*-containing nitrate reductase in eukaryotes. Studies have demonstrated via molecular analyses in field and cultured diatom populations that the gene expression and activity of nitrate reductases decreased under low Fe conditions and that the abundance of ammonium transporter (AMT) proteins increased (Timmermans et al., 1994; Marchetti et al., 2012; Nunn et al., 2013; Cohen et al., 2017). We propose that the diatoms of the IrmB had likely shifted to ammonium utilization and followed a similar nitrate reductase regulation strategy for Fe conservation. Finally, Smith et al. (2016) showed via transcriptomic analysis that the expression of the gene encoding heme oxygenase increased during Fe limitation in the diatom *Phaodactylum tricornutum*. Heme oxygenase is an enzyme that catalyzes the conversion of hemes to bilirubin thus producing Fe^2+^ and bile pigments (Frankenberg-Dinkel, 2004). Hence, the upregulation of heme oxygenase may be linked to intracellular Fe recovery processes from other porphyrins (Smith et al., 2016).

### Heme *b* as Method for Mapping Iron Limited Phytoplankton

Several methods and proxies have been proposed for the identification of Fe limited oceanic regions and phytoplankton communities. These methods include (i) the DFe:nitrate ratio (Nielsdóttir et al., 2009; Browning et al., 2017; Tonnard et al., 2018), (ii) the modified Si^∗^ tracer [Si^∗^ = (μmol silicic acid L^-1^) – (μmol nitrate L^-1^)] (Sarmiento et al., 2003; Brzezinski et al., 2015; Hogle et al., 2018), (iii) the apparent PSII photochemical efficiency (F_v_/F_m_ ratio) (Greene et al., 1994; Kolber et al., 1998), (iv) the satellite-derived quantum yield of fluorescence Φ_sat_ (Behrenfeld et al., 2009; Browning et al., 2014b), and (v) bioassay incubation experiments (Moore et al., 2006a, 2007). However, these methods can be subject to changes driven by the microbial community composition or provide an integrated picture of the nutrient status of oceanic regions, thereby missing Fe stress in phytoplankton populations that occurs over shorter temporal scales.

The parameter F_v_/F_m_ has been shown to vary among phytoplankton classes and species (Moore et al., 2005; Suggett et al., 2009), while the Si^∗^ represents the silicic acid uptake and growth only of the diatom communities under Fe limitation and cannot serve as a community-wide Fe limitation index (Hogle et al., 2018). Furthermore, the DFe:nitrate ratio provides an overall assessment of which nutrient is more likely to be limiting the phytoplankton community (Nielsdóttir et al., 2009; Moore et al., 2013). Bioassay incubation experiments successfully show the positive responses of Fe-limited phytoplankton following Fe additions (Moore et al., 2006a, 2007; Nielsdóttir et al., 2009, 2012; Ryan-Keogh et al., 2013; Browning et al., 2017). However, since the incubation bottles contain seawater of mixed microbial communities, faster growing organisms (such as diatoms) can produce a positive response to nutrient additions even when the slower growing organisms, which may be more representative of the ambient population, are not Fe limited (Price et al., 1994). Finally, although the Φ_sat_ proxy has the potential for mapping Fe limitation and has high utility in terms of spatial and temporal coverage, it also provides an integrated picture of the degree of community-level Fe limitation. In addition, Φ_sat_ is potentially subject to uncertainties associated with short and longer term changes in irradiance (Behrenfeld et al., 2009; Huot et al., 2013; Browning et al., 2014b).

**FIGURE 6:**
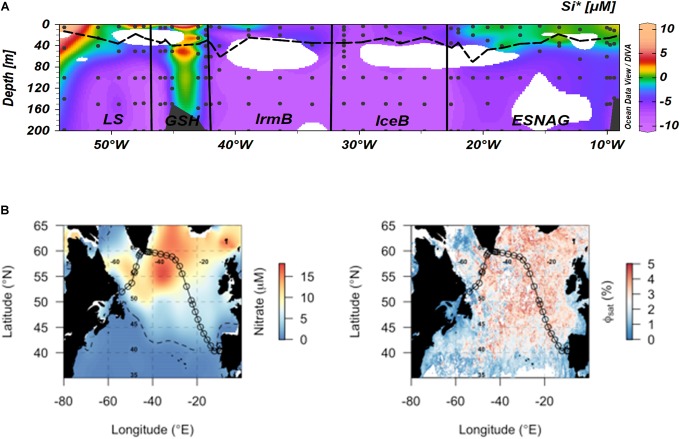
Comparison of different approaches for mapping of Fe-limited regions. **(A)** Si^∗^ tracer [Si^∗^ = (μmol silicic acid L^-1^) – (μmol nitrate L^-1^)] (Sarmiento et al., 2003). Negative values indicate potential growth limiting nutrients while positive values indicate an excess of Si(OH)_4_ after complete biological uptake of nitrate. Dashed lines indicate the Mixed Layer Depth and dots indicate the stations and depths where samples were taken. Letter annotations indicate the five oceanographic regions; Eastern Subtropical North Atlantic Ocean (ESNAG), Iceland Basin (IceB), Irminger Basin (IrmB), Greenland Shelf (GSH) and Labrador Sea (LS). **(B)** Satellite-derived quantum yield of fluorescence (Φ_sat_) averaged for May–June 2014 (right-hand side panel), calculated as in Browning et al. (2014b). Higher quantum yields (red colors) potentially indicate higher Fe stress and correlate with regions of residual nitrate (left-hand side panel) (May–June climatological surface ocean values from World Ocean Atlas; dashed line indicates the 1 μmol nitrate L^-1^ contour).

Here we compare the heme *b* method for mapping Fe-limited regions and phytoplankton communities with DFe:nitrate (Figure 3C), Si^∗^ (Figure 6A) and Φ_sat_ (Figure 6B). In general, the DFe:nitrate, Si^∗^ and Φ_sat_ methods showed good agreement and suggested that the phytoplankton communities in the IceB, IrmB and LS had the potential to be Fe-limited during the time of our study. However, we observed depleted heme *b* only in the IrmB. The discrepancy can be attributed to the fact that heme *b* represents a molecular level snapshot of the cellular activity *in situ* at the time of sampling, whilst DFe:nitrate and Si^∗^ indicate which nutrient is likely to be limiting for the phytoplankton community and Φ_sat_ integrates over a longer time scale (here 2 months) and may thus miss short term fluctuations in community Fe limitation brought about by changes in community composition. Combined use of different approaches can thus lead to a fuller picture of the factors influencing phytoplankton productivity.

We showed that changes in heme *b* relative to POC and chl *a* were linked to the stage of the phytoplankton bloom as a result of the absolute Fe requirements of each phytoplankton class. In addition, we observed large fluctuations of heme *b* relative to biomass in GSH and LS. We thus further suggest that in dynamic shelf environments phytoplankton can experience high variability in Fe stress over short temporal and spatial scales. Hence, heme *b* quotas provide further information on the level of Fe stress experienced by different phytoplankton groups and could lead to an improved understanding of the influence of Fe on community composition in productive environments.

## Conclusion

In this study, we determined heme *b* concentrations in the different biogeochemical regions of the GEOVIDE section in order to identify the factors that drive the distribution of the heme *b* Fe pool in the ocean and examine its utility as a proxy for identifying Fe-limited phytoplankton communities *in situ*. Furthermore, this study systematically examines the relationship between heme *b* abundance and phytoplankton group assemblages.

We showed that biomass variability mainly drove the heme *b* distribution and that low heme *b* quotas characterized Fe-limited phytoplankton communities (case of Irminger Basin). Our data suggest that reduction of heme *b* is not a class or species specific response to Fe limitation and that different phytoplankton groups are able to regulate heme *b* in order to optimize Fe use.

Optimization of the hemoprotein pool may be considered as an adaptation mechanism of phytoplankton to changing ambient Fe conditions. The high variability in stoichiometric ratios (e.g., Fe:C) suggest diverse mechanisms that phytoplankton employs to utilize the available nutrients during replete or limiting conditions (Moore et al., 2013). The ability to optimize Fe use and the resultant flexibility in Fe requirements needs to be accounted for when assessing areas as Fe limited or when considering the impact of Fe limitation on marine productivity and the impact of changes in Fe fluxes to the ocean in the past and future.

Direct comparison of the heme *b* method with other iron-stress biomarkers (e.g., flavodoxin, ferredoxin and IdiA) (LaRoche et al., 1996; Webb et al., 2001; Pankowski and McMinn, 2009; Saito et al., 2014) and/or bioassay incubation experiments (Moore et al., 2008; Nielsdóttir et al., 2009; Browning et al., 2014a) would reinforce the current observations that heme *b* is a good indicator of Fe limitation. Hence, heme *b* alongside other genetic (Allen et al., 2008) and proteomic (LaRoche et al., 1996; Saito et al., 2014) signatures, could be used as a reliable tool for mapping Fe limited areas and give an overview of the cellular activity of marine microbes *in situ*. Thus a broader application of heme *b* could provide information on the molecular adaptation responses of diverse phytoplankton groups to low Fe environments. This information is particularly valuable for the dynamically changing ocean and specifically in the Atlantic Ocean because of the projected altered atmospheric fluxes from the Sahara (Mahowald and Luo, 2003; Tegen et al., 2004; Jickells et al., 2005). Knowledge of phytoplankton adaptation mechanisms could thus help us predict the implications of changing Fe fluxes on key biogeochemical processes in the ocean (e.g., carbon fixation, nitrogen fixation) and their impact on global climate.

## Data Availability

Heme *b* and supporting GEOVIDE data are available on the LEFE CYBER database http://www.obs-vlfr.fr/proof/php/geovide/x_datalist_1.php?xxop=geovide&xxcamp=geovide. Gene sequences of 16S rRNA are available on the Supplementary Material while new OTU sequences are deposited to the GenBank.

## Author Contributions

EL designed the experiments. J-MB, MT, and HP carried out sampling during the expedition. EL, MT, and HP carried out the data analysis of the samples. EL carried out the data processing and statistical analyses. EL and TB prepared the figures. JLR and DD provided the sequencing data. MG, RS, EA, JLR, GS, and AB supervised the study and contributed to the interpretation of the results. EL wrote the manuscript. All authors reviewed and contributed to the writing of the manuscript.

## Conflict of Interest Statement

The authors declare that the research was conducted in the absence of any commercial or financial relationships that could be construed as a potential conflict of interest.
