# Supplementary material for: Regulation of the Phytoplankton Heme b Iron Pool During the North Atlantic Spring Bloom
Source: Front Microbiol. 2019 Jul 11;10:1566. doi: 10.3389/fmicb.2019.01566 (PMC6637849; doi:10.3389/fmicb.2019.01566)
Supplement: Supplementary file 1 [file Table_1.DOCX]

Supplementary Material

**Supplementary Table 1.** Results of Spearman’s rank correlation for heme *b*, chlorophyll *a* (chl *a*), particulate organic carbon (POC), particulate organic nitrogen (PON), nitrate and dissolved iron (DFe) overall for the GEOVIDE cruise. All correlations shown exhibited p<0.01.

|  | **Heme *b*** | **Chl *a*** | **POC** | **PON** | **Nitrate** |
| --- | --- | --- | --- | --- | --- |
| **Chl *a*** | 0.41 (153) |  |  |  |  |
| **POC** | 0.42 (165) | 0.88 (296) |  |  |  |
| **PON** | 0.40 (165) | 0.87 (295) | 0.93 (346) |  |  |
| **Nitrate** | -0.31 (84) | -0.54(136) | -0.54(151) | -0.5(151) |  |
| **DFe** | -0.07 (91) |  | -0.29(176) | -0.3(175) | 0.28 (397) |
