# Supplementary material for: Regulation of the Phytoplankton Heme b Iron Pool During the North Atlantic Spring Bloom
Source: Front Microbiol. 2019 Jul 11;10:1566. doi: 10.3389/fmicb.2019.01566 (PMC6637849; doi:10.3389/fmicb.2019.01566)
Supplement: Supplementary file 2 [file Table_2.DOCX]

Supplementary Material

**Supplementary Table 2.** List of accession numbers as determined by Chloroplast (cp) 16S rRNA sequencing. New OTUs will be deposited to the GenBank.

| AACY020080403 | EF575247 | GQ452891 | HQ671788 | JN976406 |
| --- | --- | --- | --- | --- |
| AB164407 | EF575250 | GQ452893 | HQ671819 | JN977301 |
| AB196966 | EF575303 | GQ903342 | HQ671832 | JP297615 |
| AB196967 | EU005664 | GU061402 | HQ671857 | JQ013153 |
| AB199885 | EU005687 | GU061466 | HQ671873 | JQ013163 |
| AB199887 | EU005693 | GU061468 | HQ671892 | JQ013176 |
| AB200263 | EU268091 | GU061833 | HQ671893 | JQ269285 |
| AF001655 | EU394562 | GU062154 | HQ671898 | JQ347416 |
| AF172719 | EU592338 | GU119599 | HQ671918 | JQ586285 |
| AF514855 | EU805127 | GU119711 | HQ671968 | JQ712399 |
| AM259746 | EU805239 | GU119856 | HQ672013 | JQ753195 |
| AM690833 | EU919835 | GU119858 | HQ672015 | JX015718 |
| AY135682 | EU919855 | GU119861 | HQ672034 | JX015817 |
| AY702176 | FJ002166 | GU119871 | HQ672070 | JX016173 |
| DQ395642 | FJ002174 | GU208415 | HQ672084 | JX016292 |
| EF573661 | FJ002180 | GU234935 | HQ672098 | JX016404 |
| EF573710 | FJ002195 | GU940753 | HQ672103 | JX016417 |
| EF573851 | FJ002202 | HM057648 | HQ672106 | JX016502 |
| EF573980 | FJ002206 | HM057655 | HQ672112 | JX016595 |
| EF574021 | FJ002214 | HM057658 | HQ672114 | JX016622 |
| EF574253 | FJ002228 | HM057665 | HQ672126 | JX016649 |
| EF574408 | FJ002231 | HM057706 | HQ672137 | JX016811 |
| EF574443 | FJ002238 | HM057742 | HQ672159 | JX016902 |
| EF574473 | FJ425629 | HM057746 | HQ672178 | JX016925 |
| EF574510 | FJ456816 | HM057791 | HQ672197 | JX016969 |
| EF574561 | FJ612433 | HM127595 | HQ672204 | JX017139 |
| EF574585 | FJ649257 | HM594190 | HQ672210 | JX017189 |
| EF574660 | FJ745174 | HM594191 | HQ672216 | JX297813 |
| EF574672 | FJ745242 | HQ671772 | JF272023 | JX537814 |
| EF574676 | FJ826098 | HQ203795 | JF272162 | JX537830 |
| EF574682 | FJ826213 | HQ203812 | JF272166 | JX537894 |
| EF574706 | FJ826217 | HQ203931 | JF277129 | JX537910 |
| EF574730 | FJ826341 | HQ203940 | JF277140 | JX559213 |
| EF574742 | FJ826342 | HQ203943 | JF277152 | KC425548 |
| EF574745 | FJ849120 | HQ230156 | JF344318 | KC425580 |
| EF574836 | FN396639 | HQ241996 | JF830226 | NC_007288 |
| EF574861 | FN396684 | HQ242008 | JN207202 | NC_012097 |
| EF574864 | FN396754 | HQ242211 | JN207221 | U32670 |
| EF574908 | GQ250620 | HQ242461 | JN207230 | U32671 |
| EF574962 | GQ340169 | HQ242621 | JN457994 | U70723 |
| EF575130 | GQ347890 | HQ242646 | JN625650 | X82156 |
| EF575192 | GQ348575 | HQ671746 | JN874342 |  |
