# Supplementary figures and images for: Regulation of the Phytoplankton Heme b Iron Pool During the North Atlantic Spring Bloom
Source: Front Microbiol. 2019 Jul 11;10:1566. doi: 10.3389/fmicb.2019.01566 (PMC6637849; doi:10.3389/fmicb.2019.01566)

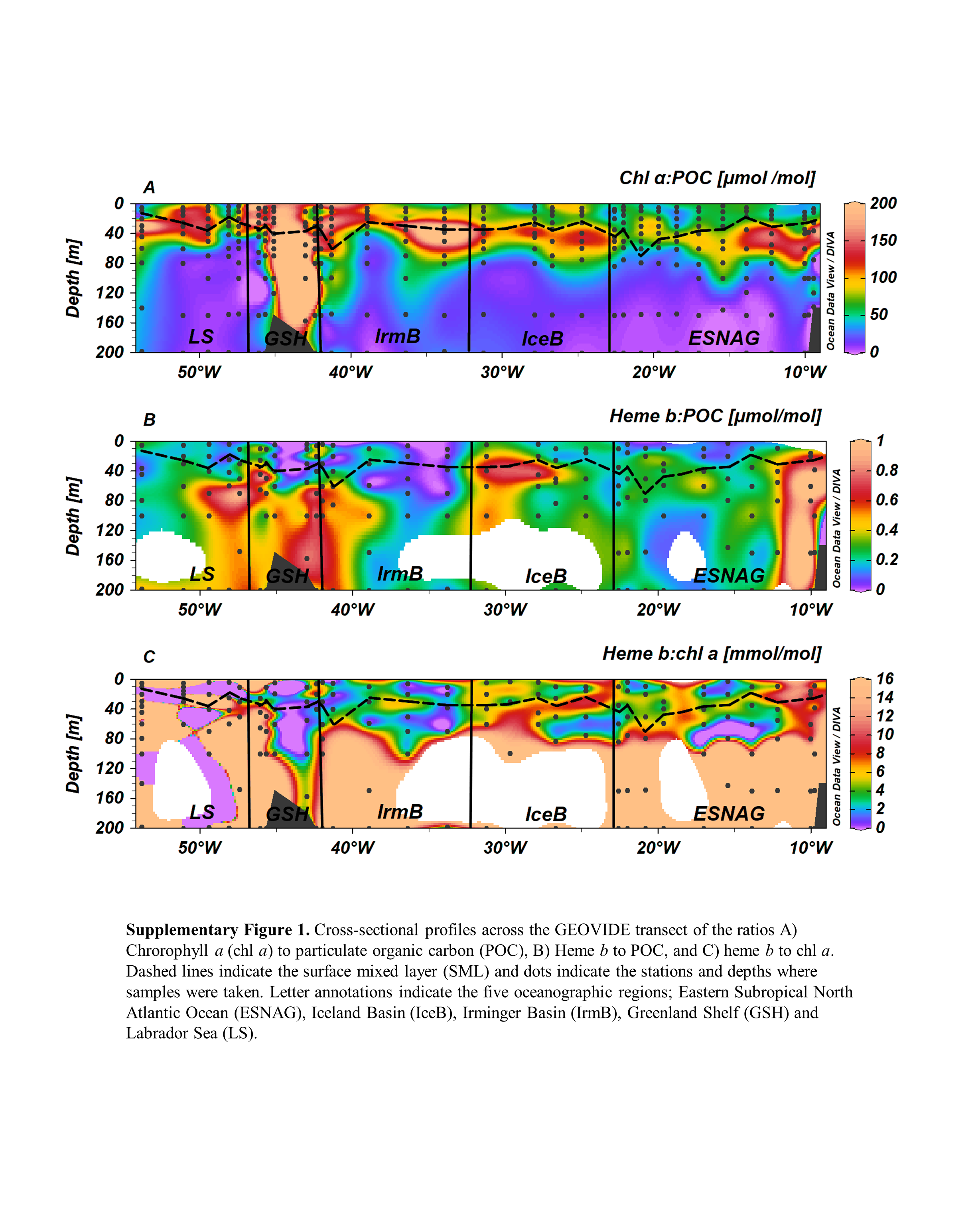

Supplement: Supplementary file 4 [file Image_1.TIF]

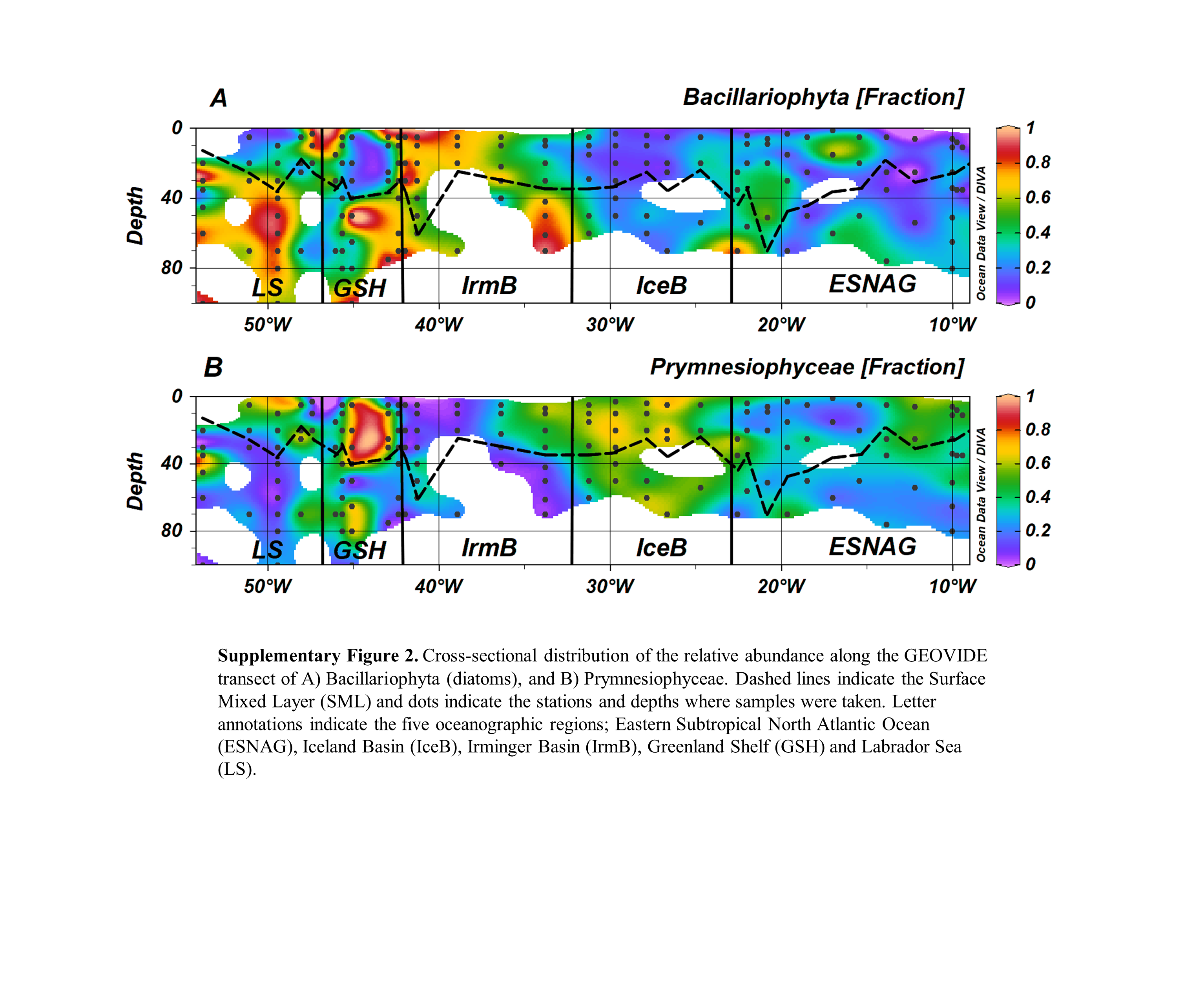

Supplement: Supplementary file 5 [file Image_2.TIF]
